# Use of Music Therapy as an Audiological Rehabilitation Tool in the Elderly Population: A Mini-Review

**DOI:** 10.3389/fnins.2021.662087

**Published:** 2021-09-16

**Authors:** Anne Sophie Grenier, Louise Lafontaine, Andréanne Sharp

**Affiliations:** ^1^CERVO Research Center, Université Laval, Québec City, QC, Canada; ^2^École d’Orthophonie et d’Audiologie, Université de Montréal, Montreal, QC, Canada

**Keywords:** music, cognition, movement, social participation, presbycusis

## Abstract

It is well known and documented that sensory perception decreases with age. In the elderly population, hearing loss and reduced vestibular function are among the most prevalently affected senses. Two important side effects of sensory deprivation are cognitive decline and decrease in social participation. Hearing loss, vestibular function impairment, and cognitive decline all lead to a decrease in social participation. Altogether, these problems have a great impact on the quality of life of the elderly. This is why a rehabilitation program covering all of these aspects would therefore be useful for clinicians. It is well known that long-term music training can lead to cortical plasticity. Behavioral improvements have been measured for cognitive abilities and sensory modalities (auditory, motor, tactile, and visual) in healthy young adults. Based on these findings, it is possible to wonder if this kind of multisensory training would be an interesting therapy to not only improve communication but also help with posture and balance, cognitive abilities, and social participation. The aim of this review is to assess and validate the impact of music therapy in the context of hearing rehabilitation in older adults. Musical therapy seems to have a positive impact on auditory perception, posture and balance, social integration, and cognition. While the benefits seem obvious, the evidence in the literature is scarce. However, there is no reason not to recommend the use of music therapy as an adjunct to audiological rehabilitation in the elderly when possible. Further investigations are needed to conclude on the extent of the benefits that music therapy could bring to older adults. More data are needed to confirm which hearing abilities can be improved based on the many characteristics of hearing loss. There is also a need to provide a clear protocol for clinicians on how this therapy should be administered to offer the greatest possible benefits.

## Introduction

Disabling hearing loss is an important problem to address among older adults. It is known to affect one-third of individuals over 65 years old (World Health Organization, 2020). The term used for age-related hearing loss in the literature is presbycusis, which includes degradation of structures in the middle ear, inner ear, and central auditory pathways due to aging (Gates and Mills, 2005). McDermott (2009) explains that all the factors that make it difficult to analyze the auditory scene in normal-hearing people are even more problematic in people with hearing loss. This is why it is not surprising that presbycusis leads not only to reduced hearing thresholds but also to difficulties to understand speech in noise, degradation of central auditory processing, and impaired localization of sounds in the environment.

The auditory image sent to the auditory cortex must be very robust so that the signal analysis (e.g., segregation, categorization) will be carried out correctly. However, in hearing-impaired individuals, this signal is degraded by hearing loss (e.g., hair cell breakage, reduced ossicle transfer function). The signal strength will therefore be reduced and distorted. In the end, the signal received at the cortex is always degraded. This results in reduced temporal and spectral resolution (Shinn-Cunningham and Best, 2008). The reduction of auditory filters makes it more difficult to analyze the auditory scene and to segregate it into its components. In addition, the harmonic structure and attack or release times appear to be less well perceived in individuals with hearing loss. These components are important for the formation of auditory objects in a short time scale (e.g., the formation of syllables during the presence of several conversations at the same time). The fine spectro-temporal structure is also less well encoded. This point can make auditory unmasking difficult, a skill necessary to isolate auditory objects from the auditory scene, for example, when it is needed to understand speech in noise.

Hearing loss in the elderly can have a variety of causes that will mostly lead to sensorineural or sometimes mixed hearing loss (Gates and Mills, 2005). There is a small number of elderly will present bone-conduction gaps in their audiogram (Nondahl et al., 2013). One way to improve hearing thresholds, for both cases, would be to find a way to directly repair the affected structures, i.e., the inner ear for the former, and the middle ear for the latter (as for example some studies try to do with stem cells, see for example Pauley et al., 2008, for a review). However, these new technologies are still in development and are not commercially available. This is why, in both situations, the most common way to improve hearing perception is by the use of hearing aids. The gain provided by hearing aids does not fully restore hearing skills. It will rather amplify environmental sounds based on hearing loss to improve audibility. However, despite sufficient hearing amplification, difficulties persist, and these are linked to persistent problems at the level of intelligibility. Several studies have shown that speech-in-noise (Marrone et al., 2008; Abrams and Kihm, 2015) and musical perception (Feldmann and Kumpf, 1988; Chasin and Russo, 2004) difficulties are recurrent complaints among hearing aid users. A way to improve auditory abilities is to combine the use of hearing aids with audiological rehabilitation. Auditory training of any kind will not be able to improve hearing thresholds due to anatomical and physiological damage limitations but will try to improve auditory processing abilities.

A lot of audiological rehabilitation programs to date are focusing mostly on auditory training (for a review, see Sweetow and Palmer, 2005). However, most audiologists are faced with patients present multiple sensory declines with vestibular disorders due to the normal aging process (dizziness, loss of balance, falls, etc.) (Hülse et al., 2019). Presbyvestibulopathy is a phenomenon of growing interest because it interferes with the maintenance of posture, balance, and movement of the elderly (Jahn, 2019). The increased risks of falls due to loss of balance in the elderly are considered a significant burden on the health system and the health of the population (World Health Organization, 2008). This other sensory loss combined with hearing loss needs to be addressed in rehabilitation approaches with the hearing-impaired elderly to enable them to have a better quality of life.

Other side effects of these sensory deprivations must be addressed in audiological rehabilitation. More and more studies are suggesting a link between hearing loss due to age, cognitive decline, and even dementia when no assistive listening devices are used (Nadhimi and Llano, 2020; Slade et al., 2020). Many leading medical organizations around the world are suggesting that age-related hearing loss can be the largest potentially preventable risk factor for dementia (Livingston et al., 2017, 2020). Once put together, all of these elements illustrate a global portrait of the limitations caused by aging of the auditory and vestibular system. These elements should be central in hearing rehabilitation programs to obtain a global portrait of the elderly and promote more accurately healthy aging.

A second important side effect of presbycusis and presbyvestibulopathy is the impact on social life of older adults. When both communication and ease of movements are altered, this can lead to impaired social participation daily to many activities (e.g., conversations, family reunions, watching television, or listening to music). Therefore, it is not surprising that another huge impact of hearing loss is on social and emotional wellbeing. As stated by World Health Organization (2020): “Exclusion from communication can have a significant impact on everyday life, causing feelings of loneliness, isolation, and frustration, particularly among older people with hearing loss.” When hearing loss is not taken care of, it will contribute to social isolation, depression, loss of self-esteem, decrease in psychological wellbeing, reduction of the ability to function in social situations, and a reduction in quality of life in general (Gates and Mills, 2005). If we add to this portrait the difficulties in maintaining posture and balance leading to limited movement, it then becomes evident that the normal aging of the inner ear can have a significant impact on the social participation of the elderly and lead to long-term psychological problems that are important to address to ensure a better quality of life for this part of the population.

Here, we question whether musical therapy could be a useful tool in audiological rehabilitation to help reverse the maladaptive plasticity linked to this sensory deprivation. It is well established that musical training can lead to functional and structural brain changes in people with normal hearing. Imaging studies have revealed that several areas of the brain, including the planum temporale, corpus callosum, the main motor area of the hand, and the cerebellum, differ in structure and size between normal musicians and non-musicians (e.g., Schlaug et al., 1995; Münte et al., 2002; Schneider et al., 2002, 2009; Herholz and Zatorre, 2012; White-Schwoch et al., 2013). These anatomical changes are associated with behavioral improvements, mainly for hearing skills. For example, improved performance has been measured for discrimination of timbre, pitch, rhythm, etc. (for a review, see Kraus and Chandrasekaran, 2010). Basic auditory processes are also improved in musicians. For example, a study by Spiegel and Watson (1984) demonstrated that musicians have better frequency discrimination compared to matched non-musician controls. Moreover, this effect seems to be correlated with the number of years of musical expertise (Kishon-Rabin et al., 2001). It is also known that musicians have better central hearing skills and that these are preserved over time (for a review, see Alain et al., 2014). In addition, it is possible to hypothesize that music not only can impact auditory abilities but also can help with social aspects and cognitive aspects. The multisensory perspective of musical training suggests that it may also lead to improvements in posture, balance, and movements. What is less clear, however, is whether learning a musical instrument at an advanced age is an effective method of rehabilitation. This question is of high relevance considering that not all individuals with hearing loss have learned a musical instrument in their lifetime. It is therefore this portion of the population that is the interest of this current literature review. The main goal of this review is to establish the impact of music therapy in the context of hearing rehabilitation of older adults. To do so, we will examine the impact of music therapy on (1) communication, (2) movements, (3) social participation, and (4) cognitive abilities of non-musician healthy old adults to determine if it could be a useful tool for audiological rehabilitation.

## Impact on Communication

It is well known that learning to play a musical instrument can lead to improvement of central auditory abilities. This is why it should be a useful way to improve central auditory abilities following hearing decline due to age. A recent study by Dubinsky et al. (2019) investigated if a group of older adults with age-related hearing loss improved their speech-in-noise and pitch discrimination abilities following musical training. Thirty-four adults with age-related hearing loss between 54 and 76 years old took part in choir lessons for 2 h per week for 10 weeks. They were compared to a do-nothing group of 29 adults matched for age and hearing loss degree. Following this training, results suggested improvement for the choir-singing group for speech-in-noise and pitch discrimination. Also, the neural representation of the pitch discrimination was measured *via* frequency following responses, an auditory evoked potential that represents the phase-locked neural activity that is synchronized to periodic and transient aspects of sound. The neural representation of pitch was also improved following musical training.

Zendel et al. (2019) also used electrophysiology to measure the speech performance in noise of 13 individuals above 65 years old participated in a 6-month piano training. They were compared to two control groups with participants matched for age, cognition, and education levels. The first group had no specific therapy (do-nothing group) (*n* = 13), and the second group (*n* = 8) followed a similar training but using video games instead of piano lessons. Participants were asked to repeat words in three conditions: No-Noise, Quiet [15 dB signal-to-noise ratio (SNR)], and Loud (15 dB SNR). Results were similar to those of Dubinsky et al. (2019): accuracy to repeat words in the loud condition was improved post-training. In addition, encephalographic measurements (EEG) showed an increase in positive cerebral electrical activity at the level of the fronto-left electrodes 200 to 1,000 ms after the presentation of a word in noise linked with the behavioral improvement found in the music group. Source analysis suggests that this activity was due to regions involved in the motor speech system. These results support the idea that musical training provides a causal benefit to hearing abilities.

With the same participants as the last study discussed, Fleming et al. (2019) performed a speech-in-noise task and made functional magnetic resonance imaging (fMRI) measurements during this behavioral task. In contradiction to their previous study, there was no improvement in measured behavior. The authors explain, however, that the nature of the task may have caused this difference. They used the hearing in noise test, which is a standard test used in audiological evaluation that consists of repeating sentences in noise. However, in this study, the participants were asked to identify the picture most related to the sentence between four pictures. This task is easier than the previous one. Also, during the EEG study, the SNR was less good and made the task more difficult, which would have avoided the ceiling effect found during the task in the fMRI. However, notable differences were found in fMRI measurements following music training. The training has led to an increase in activation of the following cortical areas when perceiving speech: the frontal cortices bilaterally, the left parietal cortex, and the right temporal bone. These areas would be associated with speech encoding. Overall, these findings support the hypothesis that musical training improve speech processing skills even when the training is started late in life.

Recently, Worschech et al. (2021) compared the impact of learning piano against listening to music as an intervention to improve speech-in-noise perception. They did their investigation using a randomized controlled trial paradigm. A total of 156 elderly with normal hearing thresholds and non-musicians were randomized into two groups: one playing piano (*n* = 74) and the other listened to cultural music (*n* = 82). Participants of the first group took part in 20 piano lesson sessions for 6 months, while the other group attended to lessons focused on listening to music for the same time period. At the end of each session, both groups completed the assigned homework for about 30 min per day. The authors measured speech reception threshold (SRT) before and after the 6-month training with the International Matrix Test (Jansen et al., 2012) that is a common clinical test used by audiologists. Globally, the results after 6 months of musical training suggested an improvement for binaural SRTs in both groups by an average of −0.14 dB. Furthermore, the piano playing group improved their left SRT by −0.46 dB in comparison to the music listening group that did not show any improvement.

Taken together, these four studies support the hypothesis that musical training started at an advanced age would be beneficial in improving speech-in-noise processing skills. Also, the first study supports that pitch discrimination threshold seems to be improved. Further investigation is needed to measure if auditory scene analysis skills in general are improved following musical training that started at an advanced age. Results for speech in noise are positive, but the amount of evidence indicates that there is a lack of research on the subject. To conclude formally would be premature, even only for that auditory ability.

## Impact on Posture and Balance

As discussed earlier, the vestibular system is also a sensory organ in the inner ear affected by normal aging. Presbyvestibulopathy can lead to problems such as difficulties to maintain balance or to perform movements that ultimately lead to increased risk of falls. Considering that learning a musical instrument is a multisensory activity and that it is easily possible to create multisensory workouts based on music, the use of music in rehabilitation could be an interesting avenue to compensate the balance and movement disorders related to normal aging. However, only two studies have looked at the question.

Auditory input has an impact on postural control and balance (e.g., Houde et al., 2016; Maheu et al., 2017a, b, 2019). It has been suggested that the auditory input can be used to compensate for vestibular loss (e.g., Maheu et al., 2019), which is a common problem among older adults (Baloh et al., 2001). By improving hearing skills, as suggested by many studies (for a review, see Kraus and Chandrasekaran, 2010), music therapy could therefore have an indirect impact on postural control and balance.

Hamburg and Clair (2003) formed a group of 16 people aged between 65 and 78 years to participate in 14 weeks of training. It consisted of a 1-h-per-week movement sequence set to music to reflect the dynamics, rhythm, timing, and phrasing of music. Balance, gait speed, and functional reach measurements were taken before training, at 5 weeks, and at the end of training. After 5 weeks, significant improvements were noted for measurements of one foot balance, walking speed, and functional reach. These results therefore suggest the potential use of music as a workout to improve balance in the elderly.

Similarly, Maclean et al. (2014) recruited 45 participants over the age of 65 years. They divided them into three distinct groups. The first group did a short workout before doing the task; they had to practice walking in rhythm to music until they were comfortable doing so. The second group also had music in the background during the walking task to accompany them but was not instructed to walk to the music. Finally, the third group did not have any training or background music during the walking task. All groups were asked to walk two times a distance of 15 m with or without background music depending on the group. They were also asked to subtract out loud from an initial number concurrently to reflect their cognitive performance during one of the two trials (single task vs. dual tasks). During the task, mean step time, velocity, number of steps, and step-time variability were measured. The results suggest that compared to the other two groups, the music trained group participants showed no dual task deficit in speed. Furthermore, after the music training, their gait stability for both the single and dual tasks improved. No improvement post-training was measured for cognitive performance demonstrating neither decline nor improvement as their gait became steadier. Furthermore, it was only the condition with training that showed this improvement. Music in the background was not sufficient to improve stability of walk.

Overall, the two studies that have looked at gait and movement following music training in older adults suggest potential improvements. The second study even suggested that a short training with music in the background may be sufficient to improve gait stability during walk in the elderly. Due to the limited amount of study and the fact that none of these has investigated whether their participants were hard of hearing or had vestibular disorders, these conclusions are, however, uncertain. When combined with evidence suggesting potential hearing improvements, these results suggest that music training could be a powerful tool for vestibular rehabilitation, but there is still a need to investigate further to provide a formal recommendation.

## Impact on Cognitive Abilities

As mentioned earlier, the aging of the world population leads to a larger number of individuals presenting cognitive decline. The prevalence of dementia is projected to double every 20 years (Lin, 2011). An increasing number of studies are suggesting a direct link between hearing loss and cognitive decline (e.g., Lin et al., 2013). A few studies have investigated musical therapy as a way to improve cognitive function in older adults without dementia.

One recent study used a category fluency task (verbal fluency ability) to investigate the impact of listening to music on cognition. Thompson et al. (2005) investigated the effect of listening to an excerpt of Vivaldi’s *The Four Seasons* on Category fluency in healthy older adults (control group) and Alzheimer’s disease patients. Thompson et al. (2005) showed that Vivaldi’s *The Four Seasons: Winter* had the largest effect on cognitive performance, which is why it was chosen for this research. For the purpose of this review, only the control group will be described. Sixteen healthy and older volunteers (5 males, 11 females, mean of age 74.94 years) were recruited for this study. Four fluency categories were used (fruits, colors, vehicles, and furniture). Participants completed two 1-min category fluency tasks while listening to music and two 1-min category fluency tasks without music. They had to name as many examples as they could among these categories. Each participant was tested individually in a quiet room. In the music condition, participants were told they would hear music. The results showed that the facilitating effect of music was small but considered significant because category fluency performance in the music condition exceeded the performance in the no-music condition.

Another study investigated the impact of music listening on verbal fluency. Mammarella et al. (2007) investigated phonemic fluency and short-term auditory memory in their study. They used Vivaldi’s music to investigate the impact of music listening on cognitive abilities during two tasks: (1) number sequence repetition (digit span, maximum of 8) and (2) to say spontaneously as much words as possible in 60 s, starting with a specific letter (phonemic fluency, maximum of 34 words). There were 24 adults, aged between 73 and 86 years (mean of 81). The participants were non-musicians and considered in good health. No training was performed before the testing. The study adopted a repeated-measures design with type of background. There were three types of background (Vivaldi’s music vs. white noise vs. no music) randomized between participants. Half of the participants were presented with the digit span first, and the other half took the fluency test first. The results for the digit span (verbal working memory) showed that the background noise had a significant effect. The mean digit span scores in the music condition were 5.25 (SD = 1.03), 4.67 (SD = 0.8) with the white noise and 4.33 (SD = 1.20) in the no-music condition. Planned comparison showed that there was a significant advantage of the music condition over the white noise and the no music condition. No difference was observed between white noise condition and the no-music condition. Concerning the phonemic fluency (verbal fluency), the results also revealed a significant effect of background noise. The mean phonemic fluency scores were 25.83 (SD = 8.23) in the music condition, 20.50 (SD = 8.63) with the white noise, and 19.62 (SD = 7.59) in the no-music condition. Planned comparisons showed the significant advantage of music over white noise and no music.

Likewise, another study used long-term training to investigate the impact of musical therapy on cognitive function. The digit span task was also used as well as many other tasks looking at the hearing/speaking dimension and visual and executive functions. Bugos et al. (2007) investigated if individualized piano instruction (IPI) can be a potential cognitive intervention to mitigate normal age-related cognitive decline in older adults. Thirty-one non-musicians aged between 60 and 85 years participated in this 9-month study. They were divided into an experimental group (*n* = 16) and a control group (*n* = 15). First, they passed a test of overall cognitive and music abilities as well as a test for working memory and executive functions in order to confirm that both groups had similar mental abilities. The experimental group received a 6-month IPI, and the control group did not. The IPI was a half-hour lesson each week, and the participants had to practice at least 3 h per week. Following the 6 months, they had 3 months without formal practice. Four subtests [digit symbol (speed, visual working memory, visuospatial processing, and visual attention), digit span (verbal working memory), block design (spatial visualization ability and motor skill), and letter number sequencing (verbal working memory)] were repeated at all three time points (before the IPI, after the 6 months, and after the 3 months of no formal practice). Also, the Trail Making Test (TMT) that assesses visual attention and task switching (Reitan and Wolfson, 1985) was administered at each of the three points. The results demonstrated that the experimental group improved over time for TMT (part B) (passing from 98.4 to 72.1 s) and digit symbols (raw score passing from 50.3 to 72.6) but not the control group. No other differences were measured between groups, suggesting improvements only for visual processing (visual working memory, visual attention, and visuospatial processing). No improvements were measured for verbal working memory in contradiction to those of Mammarella et al. (2007).

Finally, Hirokawa (2004) investigated the effect of listening to music and relaxation on arousal and verbal working memory. For this study, 15 volunteer women aged between 66 and 80 years (mean 72.7) with no dementia and no history of musicianship participated in one preliminary session in order to make sure they understood the task. They were then scheduled for three other sessions (on three different days), which took approximately 20 min each. Those four sessions were conducted in a quiet room in each subject’s respective residence. Subjects first started with an Activation–Deactivation Adjective Check List (AD ACL by Thayer, 1978 for arousal level), which is a multidimensional self-rating test constructed and extensively validated for rapid assessments of momentary activation or arousal states. Following the test, the participants were subjected to a 10-min experimental intervention in a random order. These interventions were as follows: subject preferred (a) music chosen over a specific list, (b) relaxation instructions (the modified autogenic training phrases adopted by Alice Green were used as the relaxation instructions), or (c) silence. The subjects adjusted the loudness for their comfort for music and relaxation sessions. Following the 10 min of experimental intervention, the subjects again completed the AD ACL, followed by the reading span test (Daneman and Carpenter, 1980). This test requires participants to read series of unconnected sentences aloud and to remember the final word of each sentence in a series. The subjects were presented increasingly longer sets of sentences until they failed. The results showed that music and relaxation had a positive effect on arousal, but no differences were observed on the verbal working memory performance for any of the three conditions.

Globally, the studies concerning the effect of music on older persons’ cognition abilities tend to show that there is an improvement only for verbal fluency, visual processing, and arousal. This conclusion is, however, weak because the connection between each of these studies is hard to establish because the cognitive tasks used in each study were mostly different. Additionally, the assessing conditions sometimes differed. In some of the studies, the participant was able to choose the intensity of the music, which can modify the task’s condition. There is also no information concerning the hearing status of the participants. Being in a quiet environment does not necessarily mean that the participant will perfectly understand speech, which was necessary for a lot of the tasks presented in this section. It is clear that the scientific community is keen on pursuing investigations to evaluate the impact of musical training on cognitive abilities. For example, a study protocol was recently published by James et al. (2020). Their study will follow 155 healthy elderly for 12 months to measure the impact of music training vs. musical listening on cognitive and perceptual–motor aptitudes with behavioral and fMRI tasks. Perhaps this new investigation will answer some remaining questions that will reveal the power of musical training as a rehabilitative tool.

## Impact on Social Participation

Social and emotional spheres of life of older adults are affected by hearing loss due to age (World Health Organization, 2020). Music therapy, which is a multisensory training, could be a beneficial way to improve side effects of sensory loss such as social limitations. A few studies did large-scale qualitative investigation in the population to understand how music was improving or decreasing social aspects of older adults’ life. For example, Cohen et al. (2002) integrated a questionnaire in the second phase of the Canadian Study of Health and Aging (CSHA2) to evaluate the importance of music in an elder’s life. Based on 300 respondents, results revealed that most seniors evaluated the importance of music as high. Furthermore, Hays and Minichiello (2005) measured the significance of music to elders and the contribution of music to their quality of life. After questioning 19 elders, their qualitative results suggest that music provides a connection between the elders and their memories in a way that it brings a sense of spirituality to life. These qualitative investigations suggest that music is seen as important by older adults and that it influences positively their motivation to participate in music therapy. It is well known that patient’s motivation can greatly influence the outcome of a rehabilitation process (Maclean et al., 2000).

Furthermore, some studies did an investigation on social participation in groups of older adults participated weekly to musical activities such as choirs and bands. Wise et al. (1992) studied the relationship between the dimensions of successful aging and singing in a choir. Forty-nine choir participants were compared to 49 non-musician elders matched for gender and marital status. Two main answers were obtained when asking members why they participated in a choir. Firstly, participants mentioned that they loved to sing and make music (musical component). Secondly, participants enjoyed associating with other members of the chorus (non-musical component). In accordance with these results, Hillman (2002) looked at the perceived benefit of participatory singing for seniors. A questionnaire formed of 33 items was sent to members of a choir. The answers given by participants suggest that they perceived several benefits from joining the choir. In fact, 24% of the elders mentioned an improvement of their musical abilities, and 22% reported benefits in their social life. Moreover, increased emotional wellbeing (14%), improved interest in art events (12%), and enhanced physical health (2%) were other benefits reported by the chorus members. These findings suggest a global improvement in quality of life. Furthermore, the elders rated an improvement in their social relationships. These two studies support weakly the fact that singing in a choir can have a positive impact on social life of the elderly in addition to the musical benefits and can improve globally the quality of life of older adults.

Furthermore, Coffman and Adamek (1999) asked 52 volunteer members of a wind band several questions about their quality of life and their perception of the benefits gained from participating in musical activities. First, when they were asked why they joined the band, their motivations were mostly improving their musical skills but also, in second place, to improve their social relationships. They were also asked to describe their perceived benefits from joining the band. The principal outcome was the improvement of musical skills, but the second most given answer was related to social benefits. Taken together, these three studies suggest a similar conclusion that musical activities in groups (band or choir) lead to improvement in social participation. Their findings remain limited because none of these studies compared their participants to a group of older adults doing another leisure activity or to a do-nothing group, which can bias the results.

Finally, only four studies took elders were not already participating in musical activities and introduced them to different kinds of music therapy. Vanderark et al. (1983) looked at the influence of music participation in two groups of elders following a 5-week program of music therapy. Those entered the program were at least 60 years old, and they had to be able to hear. Members were from two different private nursing homes. In the first center, 20 elders constituted the experimental group, received two 45-min sessions of the music program per week. In the second nursing home, 23 elders constituted the control group, did not receive music therapy. The association of the nursing home to either condition was random. Vanderark et al. (1983) administered three self-created questionnaires measuring five variables: self-concept, life satisfaction, quality of life, self-concept in music, and attitudes toward music. Results suggest that life satisfaction of the music program participants was improved in comparison to the do-nothing group. Also, gains for music attitude scores were higher in the experimental group than the control group. Globally, the experimental group had significant improvements for the five previous cited variables compared to the control group.

Solé et al. (2010) evaluated how participating in a choir, a music appreciation class, or a preventive music therapy session can influence the elders’ quality of life. Eighty-three elders joined one of these three activities for 9 months. Questionnaires were administrated pre-training and post-training. Globally, results from all questionnaires suggest that the three programs seemed to have positively changed the life of the participants. Also, results suggest that all music programs enhanced the quality of life of the participants.

Chan et al. (2012) conducted a randomized controlled trial to measure the effects of music on depression symptoms in elders. The research team enrolled 50 participants in their trial. In the experimental condition, the elders did 30-min listening sessions (*n* = 24). The control group (*n* = 26) did not receive any music intervention. The data were collected once a week after the music session for the experimental group (weeks 2–8) and after the rest period for the control group (weeks 1–8). The scale used to measure the level of depression symptoms was the Geriatric Depression Scale (GDS-15). The results from this study suggested that the elders with music intervention had significantly lower scores on the depression scale compared to the control group every week of the trial.

Ahessy (2016) investigated the impact of music therapy to help reduce depression symptoms and improve quality of life of seniors in a randomized controlled trial. Forty participants from a long-term residential unit and a day care center in Dubai were assigned to the control or the experimental group. The participants received a musical treatment participated in the choir for 12 weeks at a rate of 1 h per week. In opposition, the control group received the normal nursing care and they were offered, after their participation in the study, four choral sessions. Several tests were administered at the beginning and at the end of the 12-week intervention. The first questionnaire, the Cornell Scale for Depression in Dementia (CSDD), evaluated the depression level in patients with or without dementia. The Cornell Brown Scale (CBS) was the second questionnaire used to evaluate the quality of life. Also, the cognitive functioning was rated with the Mini Mental State Examination (MMSE). Finally, the experimental group could answer the choir evaluation questionnaire (CEQ) to express their thoughts about the intervention. To reduce the possible effects of the circadian rhythm and enhance the reliability of the measures, tests were administered at the same time of the day. The findings revealed that the musical intervention reduced significantly the depressive symptom scores on the CSDD for participants of the choir compared to the control group. Also, the quality of life of the seniors was significantly enhanced after the 12-week program. This improvement was not present in the control group; on the contrary, there was a decrease in their quality of life. The post-intervention results were significantly higher than (a) the results obtained before the beginning of the music therapy and (b) the results obtained for the control group after 12 weeks of standard nursing care. The cognitive functioning was also significantly improved in the experimental group compared to the control group. Regarding the benefits gained from participating in the choir (CEQ), the most answered reasons were firstly to learn new skills and secondly to maintain social relationships. These results support the importance of implementing music therapy to help reduce depression symptoms and improve quality of life of the elders.

In conclusion, a limited number of studies suggest quality of life benefits (perceived and objective) from implementing musical therapy in the life of the elders. In fact, maintaining social relationships has often been perceived as an advantage gained from participating in several music programs. Another benefit perceived from music therapy is that it helps to reduce depression symptoms in seniors. None of these studies have considered if their participants had a hearing loss, which normally accompanies aging. One study did a systematic review to evaluate the benefits of music therapy in patients were diagnosed with a chronic disease (Quach and Lee, 2017). Their results suggest that music intervention helps these patients to reduce depression symptoms. In fact, hearing loss is considered a chronic disease, but the reality of presbycusis is far from diseases like Parkinson, cancer, and diabetes considered in this study. This reflects the importance of conducting studies on (a) the impact of hearing loss on social aspects in the elderly quality of life and (b) the influence of music therapy on seniors coping with hearing loss.

## Discussion

In this review, our goal was to investigate the impact of musical training as an audiological rehabilitative tool for older adults to compensate for the difficulties linked with aging auditory and vestibular system decline and their indirect consequences leading to social and cognitive difficulties. Benefits of musical training on perceptual and cognitive skills are well documented (for a review, see Herholz and Zatorre, 2012). Furthermore, these benefits seem to persist despite the aging process (for a review, see Alain et al., 2014). Engagement in music performance over a lifetime seems to slow age-related decline for certain motor tasks (e.g., Krampe and Ericsson, 1996) and central auditory hearing abilities (e.g., Parbery-Clark et al., 2011; Zendel and Alain, 2012). Whether learning to play a musical instrument or follow a music-based training later in life can lead to similar improvements needs to be examined more closely. The main goal of this review was to establish the impact of music therapy in the context of hearing rehabilitation in older adults. We examined the literature to find studies that selected older non-musicians to participate in any form of music-based training to improve their auditory skills, gait, balance and movement, social participation, and cognitive abilities. Altogether, the big picture suggests that musical training started later in life can improve several abilities that can lead to better quality of life for the elderly. First, the three studies investigating central auditory abilities following a musical training suggest improvements for speech-in-noise perception and pitch representation (Dubinsky et al., 2019; Fleming et al., 2019; Zendel et al., 2019). Secondly, the two studies investigating gait and movements also suggest improvements for maintaining balance on one foot, walk speed, gait stability, and functional reach (Hamburg and Clair, 2003; Maclean et al., 2014). Furthermore, most studies investigating cognitive aspects following musical training suggest improvements for verbal fluency, visual processing, and arousal (Hirokawa, 2004; Thompson et al., 2005; Bugos et al., 2007; Mammarella et al., 2007). Also, social aspects of life seem to be positively influenced by participating in musical group activities for older adults. Finally, there are some major limitations to these studies that need to be addressed before making any recommendation.

One main limitation of all these studies is the lack of quantification of hearing loss. Only three studies measuring speech-in-noise abilities (Dubinsky et al., 2019; Fleming et al., 2019; Zendel et al., 2019) measured hearing thresholds of their participants. Only Dubinsky et al. (2019) used participants with typical presbycusis. The other two studies had participants were mostly normal hearing individuals or with mild hearing loss. Worschech et al. (2021) did not measure hearing thresholds of their participants; this is a major limitation of a study investigating speech-in-noise perception. All studies described above concerning gait and posture, cognitive abilities, and social abilities did not measure properly hearing threshold to quantify if their participants had hearing loss. Some are suggesting that their participants self-reported hearing well, but it is well known that it is not a good estimate of hearing loss. Hearing loss is most of the time unknown and highly stigmatized, and it takes around 10 years for an individual to realize his or her hearing difficulties (Davis L.J., 1995; Davis A., 1995). It is well known that hearing loss impacts social participation and heightens the risk of cognitive decline, and a high proportion of individuals suffering from hearing loss presents vestibular symptoms (e.g., Tien and Linthicum, 2002; Kaga et al., 2008; Cushing et al., 2013; Xu et al., 2015). This is why it should be a standard procedure to evaluate hearing of older participants when investigating any sort of abilities. Furthermore, hearing loss measurement will help provide evidence for the use of musical training as a rehabilitative tool for auditory abilities and to improve general quality of life of older adults. To do so, there is a need to correlate the degrees of hearing loss with the efficiency of musical training to guide clinicians in using this tool. For now, it is not possible to determine if only certain degrees of hearing loss can be helped *via* musical training versus, for example, more profound hearing loss where this tool would not be useful. More investigation is needed to clarify that important aspect.

Furthermore, none of these studies investigated if their participants had abnormal vestibular function. Results may have been different for all studies, not only for gait and posture, if that variable had been taken into account. There is a need to correlate the hearing and vestibular functions of participants with the results to provide a clear recommendation on whether or not to use musical therapy in audiological rehabilitation. Also, Maheu et al. (2019) suggested that auditory cues influence postural sway in individuals with vestibular dysfunction. Hearing aids can help to maintain balance for these individuals. No studies have yet asked their participants if they were using hearing aids or have reported to do so. More investigation is needed to correlate if there is a need to combine hearing aids used with musical training to obtain successful results with older individuals.

The measures of outcomes for each category of this review were disparate, which makes them difficult to compare. In the auditory abilities section, the authors were mostly focusing on speech-in-noise perception, but their methods of measuring this ability were different (word vs. phrases, different SNRs, etc.), which leads to a limited comparison. Also, whether musical training could improve other auditory analysis skills remains unknown. Both articles on gait and posture used really different outcomes too (balance on one foot, walk speed and functional reach vs. walking speed). For social aspects, the questionnaires were all different, but most of them were leading to similar conclusions about improvement of quality of life and social participation. There is still a main limitation for all social studies: they did not compare their participants to another group of older adults doing another leisure activity (not musical). Before concluding that musical training can help social participation, there is a need to investigate if just doing a leisure activity in general helps or if it is specific to music. Also, none of these studies reported if participants were doing more social activities in general after the musical training, which is an important question that needs to be addressed in further studies. Finally, the section on cognition was sparser in terms of evaluation. The batteries of evaluation were totally different from one study to another. Only one conclusion was comparable between two studies: both Thompson et al. (2005) and Mammarella et al. (2007) found improvements for verbal fluency. All other conclusions were supported by only one of the four studies described in the section. There were also huge differences between musical training among all studies. Some were focused on instrument learning or singing, which is more of a multisensory training, versus others that were more of a passive training (listening to music, exercise online, etc.). Further studies should investigate all aspects (auditory, vestibular, social, and cognition) described in this review in one study *via* a multidisciplinary evaluation. Also, there is a need to investigate which type of training is more efficient (music-based programs or learning an instrument, etc.). The duration of the training could also influence the outcome. There is also a need to investigate if improvement for each ability measured is maintained over time. Furthermore, investigation with electrophysiological measurement or imagery should be done to measure the extent of plasticity in older adults following the musical training in order to better understand neural correlates linked with their behavioral improvements. These will inform us about the extent of possibilities in terms of cortical reorganization by using musical training as a rehabilitation tool.

Similarly to this literature review, but in another area field of research, Sihvonen et al. (2017) have assessed the potential rehabilitative neurological effects of music-based intervention. In their case, they summarized results from musical therapy focusing on patients with stroke, dementia, Parkinson, epilepsy, multiple sclerosis, etc. They reached a similar conclusion to ours but for neurological rehabilitation: results tend to suggest a positive impact on patients follow a musical training program, but further controlled studies are needed to establish the efficacy of music in neurological recovery.

## Conclusion

Globally, music-based rehabilitation interventions are emerging as promising strategies. Further investigations are still needed, but all studies discussed above tend to suggest that musical training leads to improvements on all aspects targeted by this literature review (communication, posture and balance, cognitive abilities, and social participation). For future studies, researchers should measure hearing thresholds and vestibular function of their participants to evaluate if the improvements would be greater for elderly have hearing loss and/or vestibular impairments. Screening tools are available to do so, and clinicians working in rehabilitation should be encouraged to use them in order to fully understand the portrait of their patients. Also, a multidisciplinary point of view should be the focus of further research to take into account all the facets impacted by normal aging and promote aging well. The studies discussed, across all fields, used disparate musical therapies and outcome measurements. Further research is needed to identify the best musical training for each outcome because if simply listening to music can be helpful for elderly, it would be much easier to integrate into the retirement centers. However, this type of training may not be ideal for maximizing improvements considering that it does not necessarily include motor aspects compared to learning a musical instrument. Finally, neural correlates *via* EEG or fMRI should be integrated in future research to measure the possible extent of cortical plasticity as to objectify improvements.

Finally, a first step to improve the quality of life of elderly people with hearing loss would be simply to start integrating music in audiological rehabilitation programs. Also, giving access to music to elderly people in retirement homes could be a first step (e.g., to give them access to music lessons, to make musical instruments available to them, to develop choirs in centers for the elderly, or simply to give them access to portable music players to allow them to listen to the music they enjoy). In conclusion, music seems to be a powerful tool, and hearing healthcare professionals should start using it in audiological rehabilitation programs. Still, more investigations are needed to conclude the extent of benefits that this tool could bring to older adults.

## Author Contributions

All authors listed have made a substantial, direct and intellectual contribution to the work, and approved it for publication.

## Conflict of Interest

The authors declare that the research was conducted in the absence of any commercial or financial relationships that could be construed as a potential conflict of interest.

## Publisher’s Note

All claims expressed in this article are solely those of the authors and do not necessarily represent those of their affiliated organizations, or those of the publisher, the editors and the reviewers. Any product that may be evaluated in this article, or claim that may be made by its manufacturer, is not guaranteed or endorsed by the publisher.

## References

[B1] Abrams H. B. Kihm J. (2015). An introduction to MarkeTrak IX: a new baseline for the hearing aid market. *Hear. Rev.* 22:16.

[B2] Ahessy B. (2016). The use of a music therapy choir to reduce depression and improve quality of life in older adults–a randomized control trial. *Music Med.* 8 17–28. 10.47513/mmd.v8i1.451

[B3] Alain C. Zendel B. R. Hutka S. Bidelman G. M. (2014). Turning down the noise: the benefit of musical training on the aging auditory brain. *Hear. Res.* 308 162–173. 10.1016/j.heares.2013.06.008 23831039

[B4] Baloh R. W. Enrietto J. Jacobson K. M. Lin A. (2001). Age−related changes in vestibular function: a longitudinal study. *Ann. N. Y. Acad. Sci.* 942 210–219. 10.1111/j.1749-6632.2001.tb03747.x 11710463

[B5] Bugos J. A. Perlstein W. M. McCrae C. S. Brophy T. S. Bedenbaugh P. H. (2007). Individualized piano instruction enhances executive functioning and working memory in older adults. *Aging Ment. Health* 11 464–471. 10.1080/13607860601086504 17612811

[B6] Chan M. F. Wong Z. Y. Onishi H. Thayala N. V. (2012). Effects of music on depression in older people: a randomised controlled trial. *J. Clin. Nurs.* 21 776-783. 22035368 10.1111/j.1365-2702.2011.03954.x

[B7] Chasin M. Russo F. A. (2004). Hearing aids and music. *Trends Amplif.* 8 35–47. 15497032 10.1177/108471380400800202 PMC4111358

[B8] Coffman D. D. Adamek M. S. (1999). The contributions of wind band participation to quality of life of senior adults. *Music Ther. Perspect.* 17 27–31. 10.1093/mtp/17.1.27

[B9] Cohen A. Bailey B. Nilsson T. (2002). The importance of music to seniors. *Psychomusicology* 18:89. 10.1037/h0094049

[B10] Cushing S. L. Gordon K. A. Rutka J. A. James A. L. Papsin B. C. (2013). Vestibular end-organ dysfunction in children with sensorineural hearing loss and cochlear implants: an expanded cohort and etiologic assessment. *Otol. Neurotology* 34 422–428. 10.1097/mao.0b013e31827b4ba0 23370550

[B11] Daneman M. Carpenter P. A. (1980). Individual differences in working memory and reading. *J. Verbal Learn. Verbal Behav.* 19 450–466. 10.1016/S0022-5371(80)90312-6

[B12] Davis A. (1995). *Hearing in Adults: The Prevalence and Distribution of Hearing Impairment and Reported Hearing Disability in the MRC Institute of Hearing Research’s National Study of Hearing.* London: Whurr Publishers, 43–321.

[B13] Davis L. J. (1995). *Enforcing Normalcy: Disability, Deafness, and the Body.* Brooklyn, NY: Verso.

[B14] Dubinsky E. Wood E. A. Nespoli G. Russo F. A. (2019). Short-term choir singing supports speech-in-noise perception and neural pitch strength in older adults with age-related hearing loss. *Front. Neurosci.* 13:1153. 10.3389/fnins.2019.01153 31849572 PMC6892838

[B15] Feldmann H. Kumpf W. (1988). Listening to music in hearing loss with and without a hearing aid. *Laryngol. Rhinol. Otol.* 67 489–497. 3236982

[B16] Fleming D. Belleville S. Peretz I. West G. Zendel B. R. (2019). The effects of short-term musical training on the neural processing of speech-in-noise in older adults. *Brain Cogn.* 136:103592. 10.1016/j.bandc.2019.103592 31404817

[B17] Gates G. A. Mills J. H. (2005). Presbycusis. *Lancet* 366 1111–1120. 16182900 10.1016/S0140-6736(05)67423-5

[B18] Hamburg J. Clair A. A. (2003). The effects of a movement with music program on measures of balance and gait speed in healthy older adults. *J. Music Ther.* 40 212–226. 10.1093/jmt/40.3.212 14567733

[B19] Hays T. Minichiello V. (2005). The meaning of music in the lives of older people: a qualitative study. *Psychol. Music* 33 437–451. 10.1177/0305735605056160

[B20] Herholz S. C. Zatorre R. J. (2012). Musical training as a framework for brain plasticity: behavior, function, and structure. *Neuron* 76 486–502. 10.1016/j.neuron.2012.10.011 23141061

[B21] Hillman S. (2002). Participatory singing for older people: a perception of benefit. *Health Educ.* 102 163–171. 10.1108/09654280210434237

[B22] Hirokawa E. (2004). Effects of music listening and relaxation instructions on arousal changes and the working memory task in older adults. *J. Music Ther.* 41 107–127. 10.1093/jmt/41.2.107 15307814

[B23] Houde M. S. Landry S. P. Pagé S. Maheu M. Champoux F. (2016). Body perception and action following deafness. *Neural Plast.* 2016:5260671. 10.1155/2016/5260671 PMC4737455 26881115

[B24] Hülse R. Biesdorf A. Hörmann K. Stuck B. Erhart M. Hülse M. (2019). Peripheral vestibular disorders: an epidemiologic survey in 70 million individuals. *Otol. Neurotol.* 40 88–95. 10.1097/mao.0000000000002013 30289843

[B25] Jahn K. (2019). The aging vestibular system: dizziness and imbalance in the elderly. *Vestib. Disord.* 82 143–149. 10.1159/000490283 30947233

[B26] James C. E. Altenmüller E. Kliegel M. Krüger T. H. Van De Ville D. Worschech F. (2020). Train the brain with music (TBM): brain plasticity and cognitive benefits induced by musical training in elderly people in Germany and Switzerland, a study protocol for an RCT comparing musical instrumental practice to sensitization to music. *BMC Geriatr.* 20 1–19. 10.1186/s12877-020-01761-y 33087078 PMC7576734

[B27] Jansen S. Luts H. Wagener K. C. Kollmeier B. Del Rio M. Dauman R. (2012). Comparison of three types of French speech-in-noise tests: a multi-center study. *Int. J. Audiol.* 51 164–173. 10.3109/14992027.2011.633568 22122354

[B28] Kaga K. Shinjo Y. Jin Y. Takegoshi H. (2008). Vestibular failure in children with congenital deafness. *Int. J. Audiol.* 47 590–599. 10.1080/14992020802331222 18821229

[B29] Kishon-Rabin L. Amir O. Vexler Y. Zaltz Y. (2001). Pitch discrimination: are professional musicians better than non-musicians? *J. Basic Clin. Physiol. Pharmacol.* 12(Suppl. 2), 125–144. 11605682 10.1515/jbcpp.2001.12.2.125

[B30] Krampe R. T. Ericsson K. A. (1996). Maintaining excellence: deliberate practice and elite performance in young and older pianists. *J. Exp. Psychol. Gen.* 125 331–359. 10.1037/0096-3445.125.4.331 8945787

[B31] Kraus N. Chandrasekaran B. (2010). Music training for the development of auditory skills. *Nat. Rev. Neurosci.* 11 599–605. 10.1038/nrn2882 20648064

[B32] Lin F. R. (2011). Hearing loss and cognition among older adults in the United States. *J. Gerontol. A Biomed. Sci. Med. Sci.* 66 1131–1136. 10.1093/gerona/glr115 21768501 PMC3172566

[B33] Lin F. R. Yaffe K. Xia J. Xue Q. L. Harris T. B. Purchase-Helzner E. (2013). Hearing loss and cognitive decline in older adults. *JAMA Intern. Med.* 173 293–299. 23337978 10.1001/jamainternmed.2013.1868 PMC3869227

[B34] Livingston G. Huntley J. Sommerlad A. Ames D. Ballard C. Banerjee S. (2020). Dementia prevention, intervention, and care: 2020 report of the Lancet Commission. *Lancet* 396 413–446. 10.1016/s0140-6736(20)30367-6 32738937 PMC7392084

[B35] Livingston G. Sommerlad A. Orgeta V. Costafreda S. G. Huntley J. Ames D. (2017). Dementia prevention, intervention, and care. *Lancet* 390 2673–2734. 28735855 10.1016/S0140-6736(17)31363-6

[B36] Maclean L. M. Brown L. J. Astell A. J. (2014). The effect of rhythmic musical training on healthy older adults’ gait and cognitive function. *Gerontologist* 54 624–633. 10.1093/geront/gnt050 23723435

[B37] Maclean N. Pound P. Wolfe C. Rudd A. (2000). A critical review of the concept of patient motivation in the literature on physical rehabilitation. *Soc. Sci. Med.* 50 495–506. 10641802 10.1016/s0277-9536(99)00334-2

[B38] Maheu M. Behtani L. Nooristani M. Houde M. S. Delcenserie A. Leroux T. (2019). Vestibular function modulates the benefit of hearing aids in people with hearing loss during static postural control. *Ear Hear.* 40 1418–1424. 10.1097/aud.0000000000000720 30998550

[B39] Maheu M. Sharp A. Landry S. P. Champoux F. (2017a). Sensory reweighting after loss of auditory cues in healthy adults. *Gait Posture* 53 151–154. 10.1016/j.gaitpost.2017.01.015 28157577

[B40] Maheu M. Sharp A. Pagé S. Champoux F. (2017b). Congenital deafness alters sensory weighting for postural control. *Ear Hear.* 38 767–770. 10.1097/aud.0000000000000449 28504979

[B41] Mammarella N. Fairfield B. Cornoldi C. (2007). Does music enhance cognitive performance in healthy older adults? The Vivaldi effect. *Aging Clin. Exp. Res.* 19 394–399. 10.1007/bf03324720 18007118

[B42] Marrone N. Mason C. R. Kidd G. Jr. (2008). Evaluating the benefit of hearing aids in solving the cocktail party problem. *Trends Amplifi.* 12 300–315. 10.1177/1084713808325880 19010794 PMC2836772

[B43] McDermott J. H. (2009). The cocktail party problem. *Curr. Biol.* 19 R1024–R1027. 19948136 10.1016/j.cub.2009.09.005

[B44] Münte T. F. Altenmüller E. Jäncke L. (2002). The musician’s brain as a model of neuroplasticity. *Nat. Rev. Neurosci.* 3 473–478. 12042882 10.1038/nrn843

[B45] Nadhimi Y. Llano D. A. (2020). Does hearing loss lead to dementia? A review of the literature. *Hear. Res.* 402:108038. 10.1016/j.heares.2020.108038 32814645 PMC9336511

[B46] Nondahl D. M. Cruickshanks K. J. Wiley T. L. Tweed T. S. Dalton D. S. (2013). Sixteen-year change in acoustic-admittance measures among older adults: data from a population-based study. *J. Speech Lang. Hear. Res.* 56 1745–1750. 10.1044/1092-4388(2013/12-0381) 24023369 PMC3864143

[B47] Parbery-Clark A. Strait D. L. Anderson S. Hittner E. Kraus N. (2011). Musical experience and the aging auditory system: implications for cognitive abilities and hearing speech in noise. *PloS One* 6:e18082. 10.1371/journal.pone.0018082 21589653 PMC3092743

[B48] Pauley S. Kopecky B. Beisel K. Soukup G. Fritzsch B. (2008). Stem cells and molecular strategies to restore hearing. *Panminerva Med.* 50 41–53. 18427387 PMC2610336

[B49] Quach J. Lee J. A. (2017). Do music therapies reduce depressive symptoms and improve QOL in older adults with chronic disease? *Nursing* 47 58–63. 10.1097/01.nurse.0000513604.41152.0c 28538355

[B50] Reitan R. M. Wolfson D. (1985). *The Halstead-Reitan Neuropsychological Test Battery: Theory and Clinical Interpretation*, Vol. 4. Tucson, AZ: Neuropsychology Press.

[B51] Schlaug G. Jancke L. Huang Y. Steinmetz H. (1995). In vivo evidence of structural brain asymmetry in musicians. *Science* 267 699–701. 10.1126/science.7839149 7839149

[B52] Schneider P. Andermann M. Wengenroth M. Goebel R. Flor H. Rupp A. (2009). Reduced volume of Heschl’s gyrus in tinnitus. *Neuroimage* 45 927–939. 10.1016/j.neuroimage.2008.12.045 19168138

[B53] Schneider P. Scherg M. Dosch H. G. Specht H. J. Gutschalk A. Rupp A. (2002). Morphology of Heschl’s gyrus reflects enhanced activation in the auditory cortex of musicians. *Nat. Neurosci.* 5 688–694. 10.1038/nn871 12068300

[B54] Shinn-Cunningham B. G. Best V. (2008). Selective attention in normal and impaired hearing. *Trends Amplifi.* 12 283–299. 10.1177/1084713808325306 18974202 PMC2700845

[B55] Sihvonen A. J. Särkämö T. Leo V. Tervaniemi M. Altenmüller E. Soinila S. (2017). Music-based interventions in neurological rehabilitation. *Lancet Neurol.* 16 648–660. 10.1016/s1474-4422(17)30168-0 28663005

[B56] Slade K. Plack C. J. Nuttall H. E. (2020). The effects of age-related hearing loss on the brain and cognitive function. *Trends Neurosci.* 43 810–821. 10.1016/j.tins.2020.07.005 32826080

[B57] Solé C. Mercadal-Brotons M. Gallego S. Riera M. (2010). Contributions of music to aging adults’ quality of life. *J. Music Ther.* 47 264–281. 10.1093/jmt/47.3.264 21275335

[B58] Spiegel M. F. Watson C. S. (1984). Performance on frequency−discrimination tasks by musicians and nonmusicians. *J. Acoust. Soc. Am.* 76 1690–1695. 10.1121/1.391605

[B59] Sweetow R. Palmer C. V. (2005). Efficacy of individual auditory training in adults: a systematic review of the evidence. *J. Am. Acad. Audiol.* 16 494–504. 10.3766/jaaa.16.7.9 16295236

[B60] Thayer R. E. (1978). Factor analytic and reliability studies on the activation-deactivation adjective check list. *Psychol. Rep.* 42 747–756. 10.2466/pr0.1978.42.3.747 674499

[B61] Thompson R. G. Moulin C. J. A. Hayre S. Jones R. W. (2005). Music enhances category fluency in healthy older adults and Alzheimer’s disease patients. *Exp. Aging Res.* 31 91–99. 10.1080/03610730590882819 15842075

[B62] Tien H. C. Linthicum F. H. Jr. (2002). Histopathologic changes in the vestibule after cochlear implantation. *Otolaryngol. Head Neck Surg.* 127 260–264. 10.1067/mhn.2002.128555 12402002

[B63] Vanderark S. Newman I. Bell S. (1983). The effects of music participation on quality of life of the elderly. *Music Ther.* 3 71–81. 10.1093/mt/3.1.71

[B64] White-Schwoch T. Carr K. W. Anderson S. Strait D. L. Kraus N. (2013). Older adults benefit from music training early in life: biological evidence for long-term training-driven plasticity. *J. Neurosci.* 33 17667–17674. 10.1523/jneurosci.2560-13.2013 24198359 PMC3818545

[B65] Wise G. W. Hartmann D. J. Fisher B. J. (1992). Exploration of the relationship between choral singing and successful aging. *Psychol. Rep.* 70(Pt 2), 1175–1183. 10.2466/pr0.70.4.1175-1183 1496091

[B66] World Health Organization. (2008). *Global Report on Falls Prevention in Older Age.* Geneva: World Health Organization.

[B67] World Health Organization. (2020). *Deafness and Hearing Loss.* Geneva: World Health Organization.

[B68] Worschech F. Marie D. Jünemann K. Sinke C. Krüger T. H. Großbach M. (2021). Improved speech in noise perception in the elderly after 6 months of musical instruction. *Front. Neurosci.* 15:840. 10.3389/fnins.2021.696240 34305522 PMC8299120

[B69] Xu X. D. Zhang X. T. Zhang Q. Hu J. Chen Y. F. Xu M. (2015). Ocular and cervical vestibular-evoked myogenic potentials in children with cochlear implant. *Clin. Neurophysiol.* 126 1624–1631. 10.1016/j.clinph.2014.10.216 25511635

[B70] Zendel B. R. Alain C. (2012). Musicians experience less age-related decline in central auditory processing. *Psychol. Aging* 27 410–417. 10.1037/a0024816 21910546

[B71] Zendel B. R. West G. L. Belleville S. Peretz I. (2019). Musical training improves the ability to understand speech-in-noise in older adults. *Neurobiol. Aging* 81 102–115. 10.1016/j.neurobiolaging.2019.05.015 31280114

